# Structure of the catalytic domain of *Streptococcus pneumoniae* sialidase NanA

**DOI:** 10.1107/S1744309108024044

**Published:** 2008-08-20

**Authors:** Guogang Xu, Xuejun Li, Peter W. Andrew, Garry L. Taylor

**Affiliations:** a Centre for Biomolecular Sciences, University of St Andrews, St Andrews, Fife KY16 9ST, Scotland; b Department of Infection, Immunity and Inflammation, University of Leicester, Leicester LE1 9HN, England

**Keywords:** NanA, sialidases, *Streptococcus pneumoniae*

## Abstract

*Streptococcus pneumoniae* genomes encode three sialidases, NanA, NanB and NanC, which are key virulence factors that remove sialic acids from various glycoconjugates. The enzymes have potential as drug targets and also as vaccine candidates. The 115 kDa NanA is the largest of the three sialidases and is anchored to the bacterial membrane. Although recombinantly expressed full-length NanA was soluble, it failed to crystallize; therefore, a 56.5 kDa domain that retained full enzyme activity was subcloned. The purified enzyme was crystallized in 0.1 *M* MES pH 6.5, 30%(*w*/*v*) PEG 4000 using the sitting-drop vapour-diffusion method. Data were collected at 100 K to 2.5 Å resolution from a crystal grown in the presence of the inhibitor 2-deoxy-2,3-dehydro-*N*-acetyl neuraminic acid. The crystal belongs to space group *P*2_1_2_1_2_1_, with unit-cell parameters *a* = 49.2, *b* = 95.6, *c* = 226.6 Å. The structure was solved by molecular replacement and refined to final *R* and *R*_free_ factors of 0.246 and 0.298, respectively.

## Abstract 2

The structure of a catalytically active subdomain of the NanA sialidase from *S. pneumoniae* is reported to a resolution of 2.5 Å. The complex with the inhibitor Neu5Ac2en identifies the key catalytic residues and provides a platform for structure-based development of specific inhibitors.

## 1. Introduction

*Streptococcus pneumoniae* is a major human pathogen that is responsible for respiratory-tract infections, septicaemia, otitis media and meningitis. Current broad-spectrum antibiotic treatments for *S. pneumoniae* are increasingly unsuccessful owing to the emergence of drug-resistant strains (Thornsberry *et al.*, 1999 ▶). There are multivalent capsular polysaccharide vaccines available for pneumococcal disease, but their efficacy in certain high-risk groups has been questioned (Siber, 1994 ▶). More recently, a conjugate polysaccharide vaccine has been introduced successfully (Prevenar, Wyeth), but there are concerns about how readily it can be introduced globally and about its continuing efficacy. A search is therefore on for new drug and vaccine candidates for pneumococcal diseases. Several virulence factors may contribute to colonization and early infection processes (Jedrzejas, 2001 ▶). Sialidases are one key virulence factor as they remove sialic acid from host cell-surface glycans, probably unmasking certain receptors to facilitate bacterial adherence and colonization (Paton *et al.*, 1993 ▶). To date, all *S. pneumoniae* clinical isolates investigated have had prominent sialidase activities. Up to three distinct sialidases, NanA (Camara *et al.*, 1994 ▶), NanB (Berry *et al.*, 1996 ▶) and NanC, are encoded in *S. pneumoniae* genomes, with a recent study revealing NanA to be present in all clinical strains (Pettigrew *et al.*, 2006 ▶). Gene-knockout studies in mouse models have shown that NanA and NanB are essential for *S. pneumoniae* infection (Manco *et al.*, 2006 ▶).

In this paper, we report the 2.5 Å resolution X-ray crystallographic structure of the catalytic domain of *S. pneumoniae* NanA and its complex with the inhibitor 2-deoxy-2,3-dehydro-*N*-acetyl neuraminic acid (Neu5Ac2en). This provides a framework for the structure-based design of specific inhibitors of pneumococcal sialidases as potential therapeutic agents.

## 2. Experimental

Full-length NanA could be recombinantly expressed and purified, but failed to crystallize. Limited proteoloysis using trypsin, followed by mass spectrometry of the cleavage products, identified a stable sub­domain, which we designate CNanA, that spans residues 319–822 and encompasses a domain that retains the enzyme activity of the full-length NanA. The *S. pneumoniae nanA* gene in a pQE30 vector was used as a template in polymerase chain reaction (PCR) with the following primers: 5′-ACCT**CCATGG**AAGGAGCGGCTTTAACAGAGA-3′ and 5′-GGGC**CTCGAG**TTAGACCAATACTTCTGAGTCG-3′ (*Nco*I and *Xho*I restriction sites in bold). The PCR product was then ligated into the pHISTEV vector, containing six histidines and a tobacco etch virus (TEV) cleavage peptide at the N-terminus, and plasmid DNA was extracted using a Mini-Prep Kit (Promega). The plasmid was transformed into *Escherichia coli* BL21 (DE3) expression strain (Novagen) for protein expression. The transformed *E. coli* was inoculated into Luria–Bertani (LB) medium with 100 µg ml^−1^ kanamycin at 310 K. 0.5 m*M* isopropyl β-d-thiogalactopyranoside (IPTG) was added to induce CNanA expression when the optical density at 600 nm (OD_600_) of the cultures reached 0.6. Cell culture continued at 310 K for 3 h before harvesting by centrifugation at 4500*g* for 30 min at 277 K. The harvested cell pellets were resuspended in 0.1 *M* phosphate pH 7.4, 10 m*M* imidazole and sonicated with 5 × 30 s bursts. Protease-inhibitor cocktail tablets (one tablet per 25 ml extract; Roche Diagnostics) and DNAase (Sigma; final concentration 20 µg ml^−1^) were then added. The crude cell extract was centrifuged at 43 000*g* for 20 min at 277 K to remove the cell debris and the supernatant was filtered with a syringe-driven filter (0.45 µm) before starting protein purification. Soluble cell extract was loaded onto a 5 ml nickel column (GE Healthcare) and the bound protein was eluted with 300 m*M* imidazole in 0.1 *M* phosphate buffer pH 7.4. Protein purity was assessed by sodium dodecyl sulfate–polyacrylamide gel electrophoresis (SDS–PAGE) and matrix-assisted laser desorption time-of-flight mass spectrometry (MALDI–TOF). Relatively high-purity target protein was pooled for gel filtration using a 120 ml Sephacryl-200 column (GE Healthcare). The purified CNanA was dialysed against 0.1 *M* Tris–HCl pH 8.0, 150 m*M* NaCl overnight before concentration and storage.

**Figure 1:**
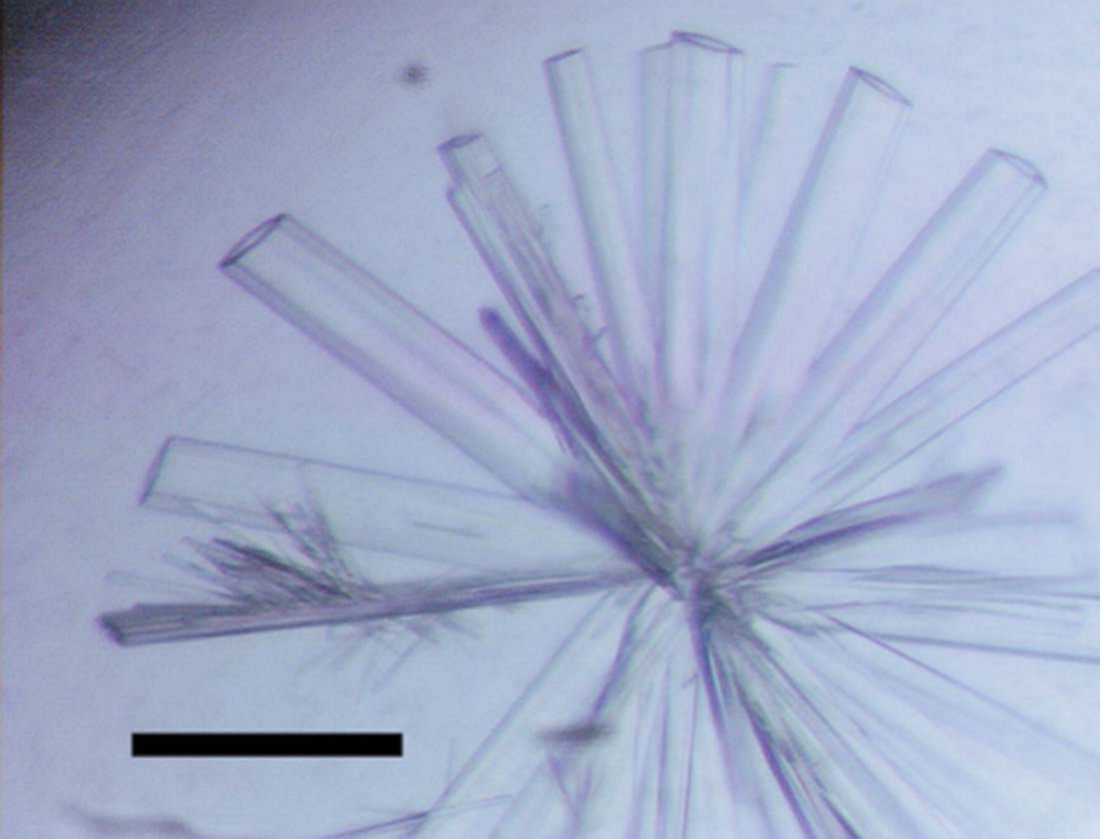
Crystals of CNanA. The scale bar represents 0.5 mm.

Purified protein was concentrated to 10.9 mg ml^−1^ for crystallization experiments using the sitting-drop vapour-diffusion method at 290 K with the commercial kits Classics (Jena Bioscience), JCSG, Nextal PEGs and Nextal pH Clear (Qiagen). Crystalline materials were observed after 3 d from condition No. 32 of Nextal PEGs [0.1 *M* MES pH 6.5, 25%(*w*/*v*) PEG 4000]. This condition was selected for crystallization optimization with crystallization drops made up of equal amounts of protein solution and reservoir solution (2 µl each). The best condition for CNanA crystallization was 0.1 *M* MES pH 6.5, 30%(*w*/*v*) PEG 4000. Crystals appeared in about 2 d and reached their maximum size after one week. The crystals have the remarkable habit of a square hollow tube (Fig. 1 ▶). Crystals of the Neu5Ac2en complex structure were grown in the presence of 10 m*M* Neu5Ac2en.

Crystals were cryoprotected by transfer for a few minutes into a solution of the crystallization buffer with 20%(*v*/*v*) glycerol before data collection at 100 K. Data were collected in-house (Rigaku-MSC MicroMax-007 HF X-ray generator and Saturn 944+ CCD detector). *HKL*-2000 (Otwinowski & Minor, 1997 ▶) was used for data processing and scaling and data-collection statistics are shown in Table 1 ▶. The catalytic domain of *Clostridium perfringens* sialidase NanI (Newstead *et al.*, 2008 ▶; PDB code 2bf6) was used to solve the CNanA structure by molecular replacement using *Phaser* (McCoy *et al.*, 2007 ▶) in the *CCP*4 suite (Collaborative Computational Project, Number 4, 1994 ▶). The asymmetric unit contains two CNanA molecules. The automated model-building wizard of the *Phenix* package (Adams *et al.*, 2002 ▶) was used to build the initial structure using the 2.5 Å resolution Neu5Ac2en complex data. This procedure built 85% of the residues with an *R* and *R*_free_ of 0.29 and 0.35, respectively. *Coot* (Emsley & Cowtan, 2004 ▶) and *REFMAC*5 (Murshudov *et al.*, 1997 ▶) were used to refine and build the final model, which was validated with *MolProbity* (Lovell *et al.*, 2003 ▶). Refinement statistics are summarized in Table 1 ▶. The first 20 amino acids, including the six-histidine tag and TEV cleavage peptide, and the last 20 amino acids are not visible in the electron-density maps; the final model consists of residues 322–791. Molecule *A* is generally well ordered, whereas molecule *B* shows disorder in its N- and C-­terminal regions. Both monomers have Neu5Ac2en bound.

## 3. Results and discussion

**Figure 2:**
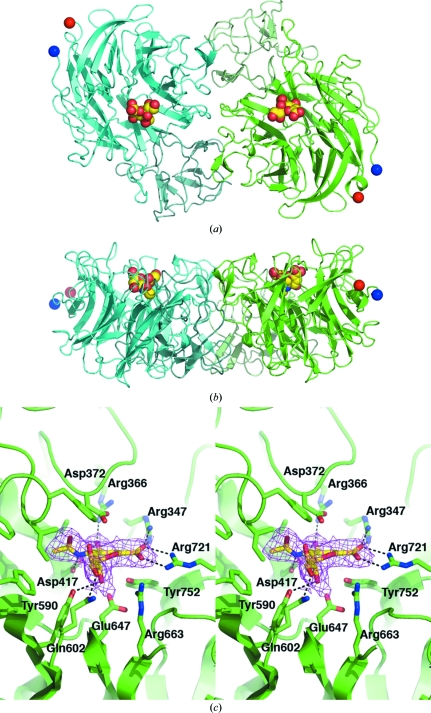
Crystal structure of CNanA. Orthogonal views of the CNanA dimer are shown in (*a*) and (*b*), where (*b*) is related to (*a*) by a 90° rotation about a horizontal axis. The N- and C-termini are indicated by blue and red spheres, respectively. Molecule *A* (green) and molecule *B* (cyan) are drawn with the inserted domains (residues 436–535) drawn in lighter shades. The inhibitor Neu5Ac2en is shown in each monomer and is drawn in space-filling mode. (*c*) Stereoview of the active site of monomer *A* showing the hydrogen-bond interactions made between Neu5Ac2en and CNanA, with only key amino acids drawn for clarity. The 2*F*_o_ − *F*_c_ electron-density map contoured at 1σ is only drawn around the inhibitor for clarity. These figures were drawn using *PyMOL* (DeLano, 2007 ▶).

The structure of CNanA shows the canonical six-bladed β-propeller fold common to all sialidases (Fig. 2 ▶). In common with the catalytic domains of *C. perfringens* NanI (Newstead *et al.*, 2008 ▶) and leech sialidase (PDB code 1sli; Luo *et al.*, 1998 ▶), CNanA has a small domain, residues 436–535, inserted between the second and third strands of the second sheet. CNanA and NanI share 41% sequence identity and the r.m.s.d. between 413 C^α^ atoms is 1.26 Å. The two monomers in the asymmetric unit are related by a noncrystallographic twofold axis, superimpose with an r.m.s.d. of 0.39 Å over 470 C^α^ atoms and bury ∼1200 Å^2^ of surface at their dimer interface. The calibrated gel filtration suggests that CNanA is predominantly a monomer in solution; however, it is possible that full-length NanA may be a dimer on the bacterial surface. The active site contains the usual catalytic residues common to all sialidases (Taylor, 1996 ▶): three arginines (Arg347, Arg663, Arg721) that interact with the carboxylate group of sialic acid, a nucleophilic tyrosine (Tyr752) that is proposed to form a covalent intermediate (Watts *et al.*, 2006 ▶; New­stead *et al.*, 2008 ▶) and its associated glutamic acid (Glu647), an aspartic acid (Asp372) that acts as an acid/base and a hydrophobic pocket that accommodates the acetamidomethyl group of sialic acid. In common with other bacterial sialidases, the hydroxyl group at C4 on Neu5Ac2en interacts with an arginine (Arg366) and an aspartic acid (Asp417). The O8 and O9 hydroxyls of the ligand’s glycerol group interact with Tyr590 and Gln602, respectively. The topology of the surface of CNanA around the location of the aglycon is flat and open, in line with the promiscuity shown by NanA towards α(2,3), α(2,6) and α(2,8) linkages (unpublished data) and in contrast to leech sialidase and *Trypanosoma cruzi trans*-sialidase, which show specificity for α(2,3)-linked sialic acids (Amaya *et al.*, 2003 ▶). Two inhibitors of the influenza virus neuramini­dase, zanamivir and oseltamivir, are currently licensed as treatments for influenza and both were developed based on the framework of Neu5Ac2en. The study reported here lays the groundwork for the potential elaboration of the core of Neu5Ac2en, particularly around the acetamido and glycerol moieties, in order to develop specific inhibitors of the pneumococcal sialidases.

## Supplementary Material

PDB reference: NanA sialidase, 2vvz, r2vvzsf

## Figures and Tables

**Table 1:** Crystallographic summary Values in parentheses are for the highest resolution shell.

Space group	*P*2_1_2_1_2_1_
Unit-cell parameters (Å)	*a* = 49.2, *b* = 95.6, *c* = 226.6
Maximum resolution (Å)	2.5 (2.54–2.50)
Unique reflections	36773
Completeness	95.2 (71.2)
*I*/σ(*I*)	27.3 (4.6)
Mosaicity (°)	0.64
Redundancy	2.9 (2.5)
*R*_merge_†	0.063 (0.248)
*V*_M_ (Å^3^ Da^−1^)	2.36
Refinement	
Protein atoms	7442
Other atoms	40 (Neu5Ac2en), 124 waters, 1 Cl^−^
Resolution range (Å)	30–2.5
*R*_cryst_‡	0.246
*R*_free_‡	0.298
Mean temperature factor (Å^2^)	
Protein, monomer *A*/*B*	29/45
Neu5Ac2en, monomer *A*/*B*	25/45
Waters	25
R.m.s.d. bond lengths (Å)	0.007
R.m.s.d. bond angles (°)	1.145
Ramachandran favoured/outliers (%)	90.5/3.7

† *R*_merge_ = 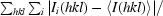 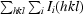.

‡ *R*_cryst_ and *R*_free_ = 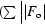 − 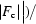 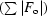; *R*_free_ was calculated for a 5% set of reflections excluded from the refinement.
